# On Plasmon Polariton Propagation Along Metallic Nano-Chain

**DOI:** 10.1007/s11468-013-9528-8

**Published:** 2013-03-28

**Authors:** Witold A. Jacak

**Affiliations:** Institute of Physics, Wroclaw University of Technology, Wyb. Wyspianskiego 27, 50–370 Wroclaw, Poland

**Keywords:** Plasmons, Metallic nano-chain, Lorentz friction, Plasmon polariton, Undamped propagation

## Abstract

The collective wave type plasmon polariton self–modes in the metallic (Au, Ag) nano-chain were determined and analyzed with respect to the nano-sphere size and chain separation parameters. At some regions for parameters, the undamped modes were identified when the interaction had been assumed as the near-field-zone dipole coupling. These modes were found on the rim of stability of the linear theory, which indicates artifact of the model of near-field coupling. Inclusion of the medium- and far-field zone contributions to dipole interaction removes, however, instability and allows for fully analytical demonstration of quenching of irradiation losses of plasmon polaritons in the chain to the level of only ohmic attenuation. The plasmon polariton dispersion and the group velocity of plasmon polariton wave packets were examined with respect to nano-sphere and chain parameters and mode polarization. Previous numerical results related to long-range plasmon polariton propagation in the chain are transparently interpreted within the analytical approach.

## Introduction

The hybridized states of surface plasmons on metal–dielectric interface with photons result in plasmon polaritons [1, 2], which are of high interest for applications in photonics and microelectronics [1, 3], in particular, for sub-diffraction transportation of converted light energy and information in metallic modified structures in nano-scale [2, 4, 5]. The propagation of plasmon polaritons along the 2D interface between metal and dielectric is a well-recognized phenomenon [6–8] widely investigated both experimentally and theoretically and also with many current and prospective applications, for sensors, plasmonic antennas, in electrochemistry, plasmon microscopy, and many other [7, 8]. The formation of plasmon polariton consists in reducing the wavelength of this mode, due to lower group velocity in comparison to the light velocity, and related concentration of the e–m field along the interface. The remarkable property is that the ideal surface plasmon polaritons have ca. 10 times lower wavelength, thus larger momentum in comparison to photons with the same energy. Therefore, it is impossible to excite plasmon polaritons by enlightening the metal surface, as well as the e–m irradiation of plasmon polaritons is quenched. Inclusion of an additional periodicity (due to grating or folding) of the surface allow, however, for matching momentum and energy conservation in interaction of plasmon polaritons with free photons.

In order to gain insight into plasmon polariton dynamics in discrete metallic planar or linear systems, there were studied arrays or chains of metallic nano-particles. Various numerical large-scale calculations of e–m field distribution in such systems were done including dipole and also multipole interaction between plasmonic oscillations in metallic components [9–13]. It is worth noticing, that the model of interacting dipoles [14, 15] was developed earlier for investigation of stellar matter [16, 17] and next it has been adopted to metallic particle systems [18, 19]. The numerical studies beyond the dipole model [9, 11] indicated that dipole model is sufficiently accurate when the particle separation is not lower than particle dimensions. Otherwise, the multipole contribution to interaction starts to be important [20]. All related analyses, mostly of numerical type, support the picture of collective plasmon polariton dynamics. Similarly to continuous planar interface between metal and dielectric, in the case of metallic nano-arrays, we encounter sub-diffraction propagation of plasmon polaritons with remarkably low attenuation. Nevertheless, the particularities of the impact of array parameters are still not clear, and visibly depend on numerical model. Therefore, the simplified but complete analytical approach to the plasmon polaritons in the metallic nano-chain would be of the value, as explicitly supplying identification of some more general tendencies and details not easy to resolve upon numerical treatments. Development of analytical approach to plasmon polaritons propagating in the nano-chain is the aim of the present paper.

To this end, we apply the Random Phase Approximation (RPA) description using a semiclassical approach suitable for a large metallic nano-sphere (with radius of several to several tens of nanometers, and with 10^5^ – 10^7^ electrons), in an all-analytical calculus version [21]. Collective dipole-type surface plasmon oscillations in the linear chain of metallic nano-spheres are next analyzed, and wave-type plasmon polariton propagation along the chain is described, beyond the previously developed near-field coupling model [4, 22, 23]. The thorough analysis of the near-field coupling between oscillating dipoles in neighboring nano-spheres, together with retardation effects for energy irradiation, indicates the occurrence of undamped propagation of plasmon waves along the chain, on the rim of instability of the system, in a certain region of values of the separation of spheres in the chain, and of the nano-sphere radii [22]. Nevertheless, this is a manifestation of a model artifact and demonstrates that not only near-field coupling plays the role in plasmon polariton dynamics in the nano-chain.

In the present paper, we include other contributions to dipole interaction in the fully retarded form, i.e, besides the near-field zone coupling also the medium- and far-field zone dipole interaction terms. This removes unphysical instability of previous near-field coupling approach and leads to the exact cancelation of the Lorentz friction in each of nano-spheres by energy income from the rest of the chain. The energy conservation arguments together with the symmetry conditions in the chain lead thus to the interpretation of this fact as to the complete quenching of irradiation losses and to the conclusion that the dissipation of plasmon polaritons is related only with the scattering effects, thus highly reduced in comparison to the single nano-sphere plasmon oscillations, especially in the case of larger nano-spheres (with radii larger than 15 nm, when for such large single nano-spheres the damping of plasmons was overwhelming by the irradiation losses [24]). This confirms the relatively long range, radiative undamped, plasmon polariton propagation in the chain, which seems to correspond with the kinetics of plasmon polariton wave packets in the finite length chain samples observed experimentally [23].

The paper is organized as follows. In the next section, the problem of plasmon damping is described in the framework of previously developed RPA semiclassical approach to plasmon oscillations in single metallic nano-sphere, being especially suitable and sufficiently accurate for description of large particles, 5 – 50 nm for radius. For irradiation losses, dominating the plasmon damping in large nano-spheres, the Lorentz friction is then analyzed, first taking into account its main linear contribution and next including also small nonlinear corrections. In the following sections, the collective propagation of plasmon oscillations along the metallic nano-chain is analyzed upon the dipole model with inclusion of near-, medium-, and far-field zones for coupling between nano-spheres and accounting for retardation effects. Particularities of an analytical calculus are shifted to the Appendix.

## Damping of Plasmons in Large Nano-Spheres

Within the RPA in semiclassical limit [21], the solution of the dynamical equation for fluctuations of local density of electrons in a metallic nano-sphere can be decomposed into two parts related to the distinct domains corresponding to the volume and surface excitations. The analysis and solution of the RPA equations have been performed in details in Ref. [21], resulting in determination of plasmon self-mode spectrum, both for volume and surface modes. Nevertheless, this RPA treatment did not account for plasmon attenuation. One can, however, include damping of plasmons in a phenomenological manner, adding an attenuation term to plasmon dynamic equations, taking advantage of their oscillatory form [21]. For the e–m wave frequency in resonance with plasmons in the metallic nano-sphere, the wavelength (then being of order of 500 nm) highly exceeds the nano-sphere size (with radius 5 – 50 nm), thus the dipole regime conditions are fulfilled. For the forcing field ${\mathbf E}(t)$, almost homogeneous over the nano-sphere (which corresponds to dipole approximation), only dipole surface mode can be excited, and the electron response resolves to a single dipole-type mode, described by the function $Q_{1m}(t)$ ($l=1$ and *m* are angular momentum numbers related to spherical symmetry). The function $Q_{1m}(t)$ satisfies the equation,

1 $$\begin{array}{lll}&&\dfrac{\partial^{2}Q_{1m}(t)}{\partial t^{2}}+\dfrac{2}{\tau_{0}}\dfrac{\partial Q_{1m}(t)}{\partial t}+\omega_{1}^{2} Q_{1m}(t) \\ &&\quad =\sqrt{\dfrac{4\pi}{3}}\dfrac{en_{e}}{m}\left[E_{z}(t)\delta_{m,0}\hspace*{-1pt}+\hspace*{-1pt}\sqrt{2}\left(E_{x}(t)\delta_{m,1}\hspace*{-1pt}+\hspace*{-1pt}E_{y}(t)\delta_{m,-1}\right)\right]\end{array}$$

where $\omega _{1}=\frac {\omega _{p}}{\sqrt {3}}$ (it is a dipole surface plasmon Mie-type frequency [21, 25]); $2/\tau _{0}$ is a damping rate; and $n_e$ and *m* are density and mass of electrons, respectively. Only this function contributes to the plasmon response to the homogeneous electric field. Thus for the homogeneous forcing field, electron density fluctuations [21],

2 $$ \delta \rho({\mathbf r},t)=\left\{ \begin{array}{l} 0,\;\; r<a,\\ \sum\limits_{m=-1}^{1}Q_{1m}(t)Y_{1m}(\Omega)\; r\geq a,\; r\rightarrow a+,\\ \end{array} \right. $$

where $Y_{lm}(\Omega )$ is the spherical function with $l=1$, and *a* is the nano-sphere radius.

For plasmon oscillations given by Eq. 2, one can calculate the corresponding dipole,

3 $$ {\mathbf D}(t)= e\int d^{3r} {\mathbf r}\delta\rho({\mathbf r},t)= \frac{4\pi}{3}e{\mathbf q}(t)a^{3} $$

where $Q_{11}(t)=\sqrt {\frac {8\pi }{3}}q_{x}(t)$, $Q_{1-1}(t)=\sqrt {\frac {8\pi }{3}}q_{y}(t)$, $Q_{10}(t)=\sqrt {\frac {4\pi }{3}}q_{x}(t)$, and ${\mathbf q}(t)$ satisfies the equation (rewritten Eq. 1),

4 $$ \left[\frac{\partial^{2}}{\partial t^{2}}+ \frac{2}{\tau_{0}} \frac{\partial}{\partial t} +\omega_1^{2}\right] {\mathbf q}(t)=\frac{en_{e}}{m} {\mathbf E}(t) $$

There are various mechanisms of plasmon damping, which could be effectively accounted for via phenomenological oscillatory-type damping term. All types of scattering phenomena, including electron–electron and electron–phonon interactions, as well contribution of the boundary scattering effect [23], cause significant attenuation of plasmons, especially important in small metal clusters. These contributions to damping time ratio are proportional to $\frac {1}{a}$ and are of lowering significance with the radius growth. In the following subsection, we argue that damping of plasmons caused by radiation losses scales conversely, as $a^3$, and for large nano-spheres, this channel dominates plasmon attenuation.

### Lorentz Friction for Plasmons

Plasmon oscillations are themselves a source of the e–m radiation. This radiation takes away the energy of plasmons resulting in their damping, which can be described as the Lorentz friction force reducing charge oscillations [26]. This damping was not included in $\tau _0$ in Eq. 4. The $\tau _0$ accounts only for scattering of electrons on other electrons, on defects, on phonons, and on nano-particle boundary, which leads to damping rate expressed by the simplified formula [23],

5 $$ \frac{1}{\tau_{0}}\simeq \frac{v_{F}}{2\lambda_{b}}+\frac{Cv_{F}}{2a}, $$

where *C* is the constant of unity order, *a* is the nano-sphere radius, $v_{F}$ is the Fermi velocity in the metal, and $\lambda _{b}$ is the electron mean free path in bulk metal (including scattering of electrons on other electrons, on impurities and on phonons [23]); e.g., for Au, $v_{F}=1.396\times 10^{6}$ m/s and $\lambda _{b}\simeq 53$ nm (at room temperature); the latter term in the formula (5) accounts for scattering of electrons on the boundary of the nano-particle, while the former one corresponds to scattering processes similar as in bulk. The other effects, as the so-called Landau damping (especially important in small clusters [27, 28]), corresponding to decay of plasmon for high energy particle–hole pair, are of lowering significance for nano-sphere radii larger than 2 –3 nm [27] and are completely negligible for radii larger than 5 nm. Note that the similarly lowering role with the radius growth is played also by electron liquid spill–out effect [29, 30], though it was of primary importance for small clusters [29, 31].

The main linear part of the electron friction caused by e–m wave emission can be described as the additional electric field [26, 32],

6 $$ {\mathbf E}_{L} = \frac{2}{3c^{3}}\frac{\partial^{3}{\mathbf D}(t)}{\partial t^{3}} $$

where *c* is the light velocity, and ${\mathbf D}(t)$ is the dipole of the nano-sphere. According to Eq. 3, one can rewrite Eq. 6 in the equivalent form,

7 $$ {\mathbf E}_{L}= \frac{2e}{3 c^{3}}\frac{4\pi}{3}a^{3}\frac{\partial^{3}{\mathbf q}(t)}{\partial t^{3}} $$

Substituting this into Eq. 4, we get,

8 $$\begin{array}{lll} &&\left[\dfrac{\partial^{2}}{\partial t^{2}}+ \dfrac{2}{\tau_{0}} \dfrac{\partial}{\partial t} +\omega_{1}^{2}\right] {\mathbf q}(t)\\&&=\dfrac{en_e}{m}{\mathbf E}(t)+\dfrac{2}{3\omega_{1}}\left(\dfrac{\omega_{1a}}{c}\right)^{3}\dfrac{\partial^{3}{\mathbf q}(t)}{\partial t^{3}} \end{array} $$

If one rewrites the above equation (for ${\mathbf E}=0$) in the form,

9 $$ \left[\frac{\partial^{2}}{\partial t^{2}} +\omega_{1}^{2}\right] {\mathbf q}(t)=\frac{\partial}{\partial t}\left[-\frac{2}{\tau_{0}} {\mathbf q}(t) +\frac{2}{3\omega_{1}}\left(\frac{\omega_{1a}}{c}\right)^{3}\frac{\partial^{2}{\mathbf q}(t)}{\partial t^{2}}\right] $$

one notes that the zeroth-order approximation (neglecting attenuation) corresponds to the equation,

10 $$ \left[\frac{\partial^{2}}{\partial t^{2}} +\omega_{1}^{2}\right] {\mathbf q}(t)= 0 $$

In order to solve Eq. 9 in the next step of perturbation iteration, one can substitute, in the r.h.s. of this equation, $\frac {\partial ^{2}{\mathbf q}(t)}{\partial t^{2}}$ by $-\omega _{1}^{2} {\mathbf q}(t) $ (acc. to Eq. 10).

Therefore, if one assumes the above estimation, i.e., $ \frac {\partial ^{3}{\mathbf q}(t)}{\partial t^{3}}\simeq -\omega _{1}^{2} \frac {\partial {\mathbf q}(t)}{\partial t}$, then one can include the Lorentz friction (its main linear part) into the renormalized damping term:

11 $$ \left[\frac{\partial^{2}}{\partial t^{2}}+ \frac{2}{\tau} \frac{\partial}{\partial t} +\omega_{1}^{2}\right] {\mathbf q}(t)=\frac{en_{e}}{m}{\mathbf E}(t) $$

where

12 $$ \frac{1}{\tau}=\frac{1}{\tau_{0}}+\frac{\omega_{1}}{3}\left(\frac{\omega_{1} a}{v}\right)^{3}\simeq \frac{v_{F}}{2\lambda_{B}}+\frac{Cv_{F}}{2a}+ \frac{\omega_{1}}{3}\left(\frac{\omega_{1} a}{c}\right)^{3} $$

The renormalized damping, sharply sensitive to the nano-sphere radius, causes a change in the shift of self-frequencies of free surface plasmons, $\omega _1'=\sqrt {\omega _{1}^{2}-\frac {1}{\tau ^{2}}}$, which can be compared with the experimental observations for various nano-sphere radii [24]. The radius-dependent shift of the resonance resulting due to strong irradiation-induced plasmon damping has been confirmed experimentally [24] by measurement of light extinction in colloidal solutions of nano-particles with different size (it has been done [24] for Au, 10 – 80 nm, and Ag, 10 – 60 nm). These measurements clearly support the $a^3$ plasmon damping scaling, as described above for the far-field zone radiation losses in a dielectric surrounding.

One can verify also that the above-calculated Lorentz friction contribution to plasmon damping is exactly equal to the energy transfer to the far-field zone (which can be expressed by the Poynting vector and via comparison with the energy loss of plasmon oscillations). In this way, we have arrived [21, 24] at the same formula for damping time rate as given by Eq. 12. This indicates that accounting for the Lorentz friction in the form of (6) reproduces radiation type energy transfer from plasmon oscillations to the far-field zone, i.e., in the vacuum or in the dielectric surroundings. If, however, the another charged system is located in the vicinity of the nano-sphere, the situation changes. For instance, in the case when the nano-sphere is deposited on the semiconductor surface, the near-field coupling of plasmons with semiconductor band electrons results in a very effective energy transfer to the semiconductor substrate and quick damping of plasmons. Similarly, the presence of other nano-spheres in the chain considerably changes radiative attenuation rate of plasmon oscillations in each nano-sphere.

## Collective Plasmon Wave-Type Propagation along the Nano-Chain

In the case of the metallic nano-chain, one has to take into account the mutual affecting of nano-spheres in the chain. Assuming that we deal with the dipole $\mathbf {D}(t)$ in the sphere located in the point $\mathbf {r}$, then in the other place $\mathbf {r}_0$ (the vector $\mathbf {r}_0$ is fixed to the end of $\mathbf {r}$), this dipole causes electric and magnetic fields in the form as follows (including electro-magnetic retardation) [26, 32]:for $\omega $-Fourier component of the electric field,

13 $$\begin{array}{rll} \mathbf{E}_{\omega}&=&\mathbf{D}_{\omega}\left(\frac{(\omega /c)^{2}}{r_0}+\frac{i\omega /c}{r_{0}^{2}}-\frac{1}{r_{0}^{3}}\right) e^{i\omega r_{0/c}}\\ && +\mathbf{n}_{0}(\mathbf{n}_{0}\cdot \mathbf{D}_{\omega}) \left( -\frac{(\omega /c)^{2}}{r_{0}}-\frac{i3\omega /c}{r_{0}^{2}}+\frac{3}{r_{0}^{3}}\right) e^{i\omega r_{0/c}}\\ \end{array} $$

and for the magnetic field Fourier component,

14 $$ \mathbf{B}_{\omega}=i \omega/c (\mathbf{D}_{\omega}\times \mathbf{n}_{0}) \left(\frac{i\omega/c}{r_{0}}-\frac{1}{r_{0}^{2}}\right) e^{i\omega r_{0/c}} $$

where $\mathbf {n}_{0}=\frac {\mathbf {r}_{0}}{r_{0}}$.

### Near-Field Zone Approximation of Dipole Interaction in the Chain

To examine the role of the terms corresponding to the near-field zone (denominator with $r_{0}^{3}$), medium-field zone (denominator $r_{0}^{2}$), and far-field zone (denominator with $r_{0}$), let us first confine ourselves to the near-field zone dipole-type coupling. In this approximation, only electric field is present, and its form resolves itself to the static dipole-field formula,

15 $$ \begin{array}{l} \mathbf{E}(\mathbf{r},\mathbf{r}_{0},t)=\frac{1}{{r_{0}}^{3}}\left\{3\mathbf{n}_{0}\left(\mathbf{n}_{0} \cdotp\mathbf{D}\left(\mathbf{r},t-\frac{r_{0}}{c}\right)\right) -\mathbf{D} \left(\mathbf{r},t-\frac{r_{0}}{c}\right)\right\}\\ \end{array} $$

This allows for writing out the dynamical equation for plasmon oscillations at each nano-sphere of the chain, which can be numbered by integer *l* (*d* will denote the separation between nano-spheres in the chain, $d>2a$; vectors $\mathbf {r}$ and $\mathbf {r}_{0}$ are collinear, if the origin is associated with one of nano-spheres in the chain). Note additionally that, as it follows from the numerical studies [9, 19], the dipole approximation of plasmon interaction in the nano-sphere chain is sufficiently accurate for $d{} >{}3a$, when multipole interaction contribution can be neglected.

This dynamical equation attains the form,

16 $$\begin{array}{lll} &&\ddot{R}_{\alpha}(ld, \omega_{1} t) +R_{\alpha}(ld,\omega_{1}t) \\ &&\quad=\sigma_{\alpha}\frac{a^{3}}{d^{3}} \sum\limits_{m=-\infty,m\neq l}^{\infty} \frac{R_{\alpha}\left(md,\omega_{1} t-\frac{\omega_{1} d|l-m|}{c}\right)}{|l-m|^{3}} \\&& \qquad-\frac{2}{\tau_{0} \omega_{1}}\dot{R}_{\alpha}(ld,\omega_{1} t) +\frac{e}{ma{\omega_{1}}^{2}}E_{\alpha}(ld,\omega_{1} t) \end{array} $$

where $R_{\alpha }(\omega _{1} t)=\frac {D_{\alpha }(\omega _{1}t/\omega _{1})}{eN_{e} a}$, dots indicate derivatives with respect to dimensionless $t'=\omega _{1} t$ and $\sigma _{\alpha }=\left \{\begin {array}{l}-1,\;for\;\alpha =x(y),\\2,\;for\;\alpha =z,\end {array}\right .$ is introduced to distinguish two polarizations of oscillations with respect to the chain orientation. The index $\alpha $ enumerates polarizations, longitudinal and transversal ones with respect to the chain orientation (*z* axis). The first term of the r.h.s. in Eq. 16 describes the dipole-type coupling in near-field zone between nano-spheres, and the other two terms correspond to contribution due to plasmon attenuation (including the Lorentz friction linear term which can be next accounted for as described in the previous paragraph). Note that the similar approach, including only the near-field dipole coupling in the chain, was utilized also by Atwater group [4, 23].

The summation in the first term of the r.h.s. of the Eq. 16 can be explicitly performed in the manner as presented in Ref. [22], if one changes to the wave vector picture, taking advantage of the chain periodicity (in analogy to Bloch states in crystals with the reciprocal lattice of quasi-momentum), i.e.,

17 $$ R_{\alpha}(ld,t)=R_{\alpha}(k,t)e^{\mp ikld} , 0\leq k \leq\frac{2\pi}{d} $$

where $\mp k$ correspond to two possible orientations of phase velocity (time factor is assumed as $e^{-i\omega t - t/\tau }$). Thus, the Eq. 16 can be rewritten (cf. Appendix) in the following form (the Lorentz friction term was represented similarly as in Eq. 11),

18 $$ \ddot{R}_{\alpha}(k_{\alpha},t\omega_{1})+ \tilde{\omega}_{\alpha}^{2} R_{\alpha}(k_{\alpha},t\omega_{1}) =-2\dot{R}_{\alpha}(k_{\alpha},t\omega_{1})\frac{1}{\tau_{\alpha} \omega_{1}}\\ $$

where

19 $$ \tilde{\omega}_{\alpha}^{2}= \left(\frac{\omega_{\alpha}}{\omega_{1}}\right)^{2}=1-2\sigma_{\alpha}\frac{a^{3}}{d^{3}}cos(k_{\alpha}d) cos\left(\frac{\omega_{p}d}{c\sqrt{3}}\right), $$

20 $$\begin{array}{rll} \frac{1}{\tau_{\alpha}\omega_{1}}&=&\frac{1}{\tau_{0}\omega_{1}}+\left(\frac{1}{3}+ \frac{\sigma_{\alpha}}{12}\right)\left(\frac{\omega_{pa}}{c\sqrt{3}}\right)^{3} +\sigma_{\alpha}\frac{a^{3}}{d^{3}}\left(\frac{\omega_{p} d}{c\sqrt{3}}\right)\\ &&- \left[\frac{\pi^{2}}{6}-\frac{\pi k_{\alpha}d}{2}+\frac{(k_{\alpha}d)^{2}}{4}\right]. \end{array} $$

**Fig. 1:**
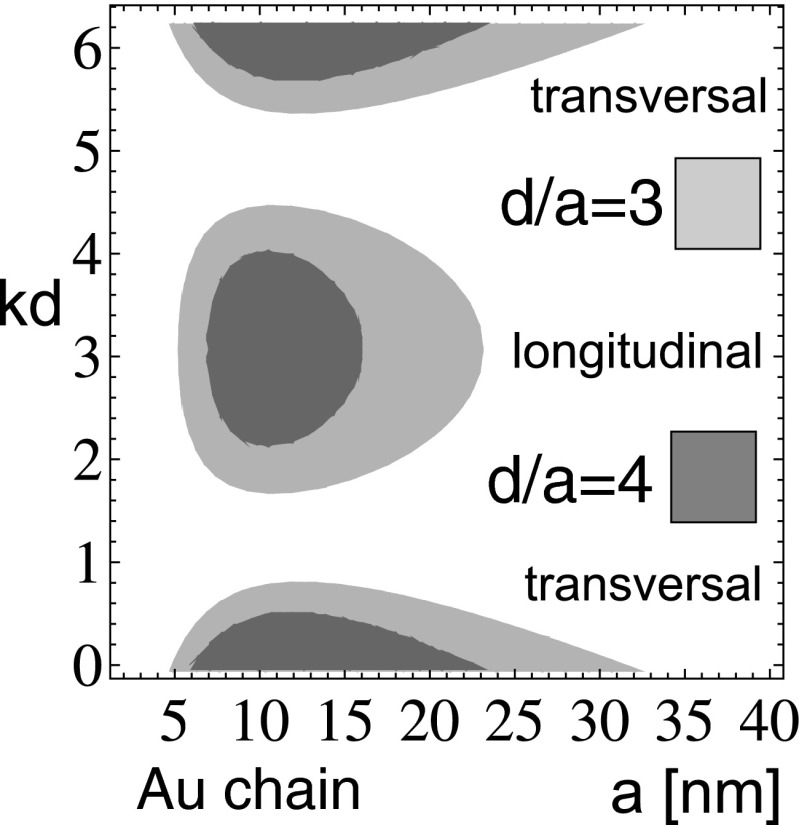
The regions with negative value of damping rates for plasmon polaritons in the chain (for longitudinal polarization and for transversal one) when only near-field dipole coupling between nano-spheres was included (they shrink with $d/a$ growth and disappear, longitudinal (transversal) for $d/a> 6(7)$; these regions completely disappear, longitudinal modes for $3.5> a>35$ nm and transversal modes for $3.5> a>48$ nm, for Au nano-spheres)

Formula (20) expresses the attenuation rates for both polarizations. Two components of Eq. 20, for $\alpha =x(y)$ and *z*, give these damping rates explicitly, and one can notice a remarkable property, that the effective attenuation rates could change their signs depending on values for *d*, *a*, and *k*. In Fig. 1, the regions of negative value for damping rates are marked (for both polarizations). These regions are shrinking with the growth of $d/a$ and with the growth of *a* itself. For *a* larger than some critical value, these regions disappear: longitudinal modes for $a>35 $ nm and transversal modes for $a>48$ nm (for Au nano-spheres).

Using Eq. 19, one can calculate the group velocity of the plasmon polariton mode packet upon the near-field coupling approximation, in the following form,

21 $$ v_{\alpha}=\frac{d\omega_{\alpha}}{dk}=\omega_{1} \frac{\sigma_{\alpha}a^{3}\sin(kd) \cos\left(\frac{\omega_{1}d}{c}\right)}{d^{2}\sqrt{1-2\sigma_{\alpha}\frac{a^{3}}{d^{3}}\cos (kd)\cos\left(\frac{\omega_{1}d}{c}\right)}} $$

From this formula, it follows that the group velocity of the undamped or damped wave type collective plasmon excitation may attain different values depending on *a*, *d*, and *k*, as it is depicted in Fig. 3 (upper). With growing *a*, this velocity grows proportionally and diminishes with the separation of nano-spheres in the chain as $\sim (d/a)^{-2}$. In Fig. 4 (upper), the dispersion of collective plasmons in the chain in the same near-field coupling approximation is plotted versus the wave vector and the separation of nano-spheres in the chain (for Au nano-spheres with the radius, $a=10$ nm).

For the positive attenuation rate, one can expect ordinary damped plasmon polariton propagation, while in the case of the negative damping rate, the solution behaves differently revealing instability of the linear theory. The negative value of the damping rate indicates instability of the system, which is, however, unphysical artifact of the model, in view of energy conservation constraints. In other words, the continuous losses of plasmon oscillation energy due to scattering and irradiation would be instantly recovered by e–m influence of other nano-spheres in the chain. When this income would prevail losses, then the packet of corresponding modes would propagate without damping. The model of the near-field coupling admits such unphysical scenario, for losses lower than a certain threshold. For nano-spheres too small (with radii lower than ca. 3.5 nm [depending on the constant *C* in the formula for scattering damping rate], for Au particles), the scattering attenuation on particle boundaries, $\sim \frac {C v_F}{2a\omega _1}$, is too high, similarly as irradiation losses, $\sim \left (\frac {1}{3} +\frac {\sigma _{\alpha }}{12}\right )\left (\frac {\omega _{pa}}{c\sqrt {3}}\right )^3$, are above the required threshold for $a>35(48)$ nm for longitudinal (transversal) modes. Thus outside the regions $3.5<a<35(48)$ nm undamped longitudinal (transversal) modes in near-field coupling approximation do not occur regardless to separation in the chain. The chain separation, $d/a$, influences the range of this undamped propagation additionally, which is illustrated in Fig. 1.

### Medium- and Far-field Corrections to Near-field Dipole Interaction in the Chain

The above-described instability of the near-field coupling approach indicates that some other effects, essential for plasmon polariton attenuations, were not taken into account. The required correction can be linked with the form of dipole influence of other nano-spheres assumed previously in the form (15). To examine this instability of the near-field zone approach, the medium-field zone and far-field zone contributions must be taken into account. Thus, using the formula (13), one arrives at the inter-dipole interaction contribution to the oscillator equation on the *l*th nano-sphere (cf. Appendix),

22 $$\begin{array}{lll} && e^{\mp ikld} e^{-i\omega t} e^{-\tau t} R_{\alpha} (k)a^{3}\sum\limits_{n=-\infty, n\neq l}^{\infty} e^{\pm iknd}\\ &&\quad (F_{1}(|n|) +i F_{2}(|n|)) e^{i|n|\omega d/c} \end{array} $$

or

23 $$\begin{array}{lll} && e^{\mp ikld}e^{-i\omega t}e^{-\tau t} R_{\alpha} (k) 2a^{3}\sum\limits_{n=1, n\neq l}^{\infty} cos(knd)\\ &&\quad(F_1(|n|) +i F_{2}(|n|)) e^{i|n|\omega d/c} \end{array} $$

where

24 $$ F_{1}=\left\{ \begin{array}{l} \frac{(\omega/c)^{2}}{|n|d} -\frac{1}{|n|^{3} d^{3}}, \; for\;\; \alpha=x(y),\\ [8pt] \frac{2}{|n|^{3}d^{3} }, \;for\;\; \alpha=z \end{array}\right. $$

and

25 $$ F_{2}=\left\{ \begin{array}{l} \frac{\omega/c}{|n|^2d^2}, \; for\;\; \alpha=x(y),\\ [8pt] -\frac{2 \omega/c}{|n|^{2}d^{2}}, \;for\;\; \alpha=z \end{array}\right. $$

Employing the above formulae, one can write out the contributions to the real and imaginary parts of the dynamical equation, which give corrections to the frequency $\omega _{\alpha }$ and to the damping rate $\tau _{\alpha }$, respectively,

26 $$\begin{array}{lll} &&2a^{3}\sum\nolimits_{n} cos(knd) F_{1} cos(n\omega d/c)\\ &&\qquad - 2 a^{3}\sum_{n} cos(knd)F_{2} sin(n\omega d/c),\\ && -2a^{3}\sum_{n} cos(knd) F_{1} sin(n\omega d/c)\\ &&\qquad - 2a^{3}\sum_n cos(knd) F_{2} cos(n\omega d /c) \end{array} $$

These contributions to the frequency of plasmon polariton oscillations and to the corresponding damping term have the explicit form, for the longitudinal polarization ($\alpha = z$), corrections to the frequency and to the damping rate,

27 $$\begin{array}{lll} && a^{3} \left[2\sum\limits_{n} cos(knd) \frac{2}{n^{3}d^{3}} cos (n\omega d/c)\right.\\ && \qquad +2\sum\limits_{n} cos(knd)\left.\frac{2\omega/c}{n^{2}d^{2}} sin(n\omega d/c)\right] \end{array} $$

and

28 $$\begin{array}{lll} &&a^{3} \left[-2\sum\limits_{n} cos(knd) \frac{2}{n^{3}d^{3}} sin(n\omega d/c)\right.\\ &&\qquad+2\sum\limits_{n} cos(knd)\left.\frac{2\omega/c}{n^{2}d^{2}} cos(n\omega d/c)\right] \end{array} $$

and similarly for the transversal polarization ($\alpha =x(y)$),

29 $$\begin{array}{lll} && a^{3} \left[-2\sum_{n} cos(knd) \frac{2}{n^{3}d^{3}} sin(n\omega d/c)\right. \\ &&\qquad +2\sum_{n} cos(knd)\left.\frac{2\omega/c}{n^{2}d^{2}} cos(n\omega d/c)\right] \end{array}$$

and

30 $$ \begin{array}{l} a^{3} \left[-2\sum_{n} cos(knd) \left( \frac{(\omega/c)^{2}}{nd}-\frac{1}{n^{3}d^{3}}\right) sin(n\omega d/c)\right.\\ \qquad\left.-2\sum_{n} cos(knd) \frac{\omega /c}{n^{2}d^{2}} cos(n\omega d/c)\right].\\ \end{array} $$

To proceed with estimation of these contributions to the oscillator equation, one can apply perturbation iterative method of solution which in the first step resolves to substitution of $\omega $ with free frequency $\omega _{1}$ in the r.h.s. of the oscillatory equation, in the manner as described in the Appendix. The sums in the formulae for damping ratio contributions can be performed accurately [33] (for an arbitrary $\omega $. including $\omega =\omega _{1}$), cf. Appendix. In the result, one obtains (for $kd-\omega _{1} d/c>0$ and $kd+\omega _{1} d/c<2\pi $), for transversal polarization,

31 $$\begin{array}{lll} &&\frac{a}{d} \left(\frac{\omega_{1} a}{c}\right)^{2} (\omega_{1} d /c)+\frac{a^{2}}{d^{2}} \left(\frac{\omega_{1} a}{c} \right)\\ &&\quad\; \times \left[\pi^{2}/3 -\pi kd +(kd)^{2} /2+(\omega_{1} d/c)^{2}/6\right]\\ &&\quad\; -\frac{a^{2}}{d^{2}} \left(\frac{\omega_{1} a }{c}\right) \left[\pi^{2}/3 -\pi kd +(kd)^{2} /2 +(\omega_1 d/c)^{2} /2\right]\\ &&\qquad\; ={}\left(\frac{\omega_{1} a}{c}\right)^{3}{}-1/3 (\omega_{1} a /c)^{3}{}=2/3\left(\frac{\omega_{1} a}{c}\right)^{3}, \end{array} $$

and for longitudinal polarization,

32 $$ \begin{array}{l}-\dfrac{a^{3}}{d^{3}}2 \left(\dfrac{\omega_{1} d}{c}\right) \left[\pi^{2}/3 -\pi kd +(kd)^{2} /2 +(\omega_{1} d/c)^{2} /6\right] \\+\dfrac{a^{2}}{d^{2}} 2\left(\dfrac{\omega_{1} a }{c}\right) \left[\pi^{2}/3 -\pi kd +(kd)^{2} /2 +(\omega_{1} d/c)^{2} /2\right] \\=2/3 \left(\dfrac{\omega_{1} a}{c}\right)^{3}.\\\end{array} $$

(note again that in the above formulae substituting of $\omega _{1}$ with $\omega _{\alpha }$ gives their accurate form).

We see that for both polarizations, the instability disappeared (i.e., the contribution to damping rate does not change its sign, as it was in the case of sole near-field zone contribution [22]). The term with the denominator $r^{2}$ (imaginary) [for transversal polarization also contributes the real term with the denominator *r*] exactly cancels the previous instable contribution of the term with the denominator $r^{3}$ (real).

**Fig. 2:**
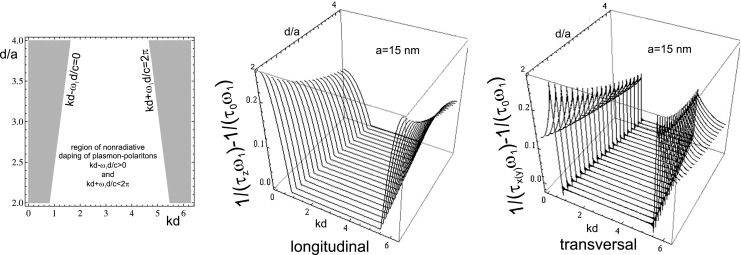
The region with completely quenched radiative losses of plasmon polaritons (PP) (*left*), damping rates beyond the pure scattering contribution (i.e., $\frac {1}{\tau _{\alpha }\omega _{1}} - \frac {1}{\tau _{0}\omega _{1}}$) for both polarizations (*right*); near-, medium-, and far-field dipole coupling included; small oscillations in the graph for transversal polarization are caused by slowly convergent $\sum _{i} sin(ix)/i$ (there were taken 300 elements of this sum), this sum gives also discontinuity finite step on the radiatively undamped region border

Simultaneously, the Lorentz friction $\big (2/(\tau \omega _{1}) = 2/3\left ( \frac {\omega _{1} a}{c}\right )^{3}\big )$ is completely canceled by the above-calculated contribution to energy income from other nano-spheres in the chain, for both polarizations, but only when $kd - \omega _{1} d/c>0$ and $kd+\omega _{1} d/c<2\pi $, what is illustrated in Fig. 2. In other words, plasmon polaritons do not irradiate energy and dissipation of energy is only due to electron scattering—this confirms the previous numerical observations [9, 11, 12]. This perfect cancelation of the irradiation losses is exact in the first step of iterative perturbation procedure, when in both terms, corresponding to Lorentz friction and to energy income from other nano-spheres, the frequency is assumed as $\omega _{1}$. In order to answer the question, whether this perfect quenching of radiation holds in general, i.e., for resonance frequency in the chain, $\omega _{\alpha }$, one can notice that the accurate energy income is still given by Eqs. 31 and 32 with $\omega _{1}$ changed for $\omega _{\alpha }$. Simultaneously, the linear part of the Lorentz friction attains the exact form, $2/3\left (\frac {\omega _{\alpha }a}{c}\right )^{3}$ (due to equality, $\frac {\partial ^{3}R_{\alpha }}{\partial t^{3}}=-\omega _{\alpha }^{2} \frac {\partial R_{\alpha }}{\partial t}$, which is satisfied for $R_{\alpha }\sim e^{-i \omega _{\alpha } t}$; the third co–factor with $\omega _{\alpha }$, in the expression for the Lorentz friction results from $1/\tau _{\alpha }= \omega _{\alpha }/(\tau _{\alpha }\omega _{\alpha })$). Thus one can argue that the cancelation is perfect also in this case.

**Fig. 3:**
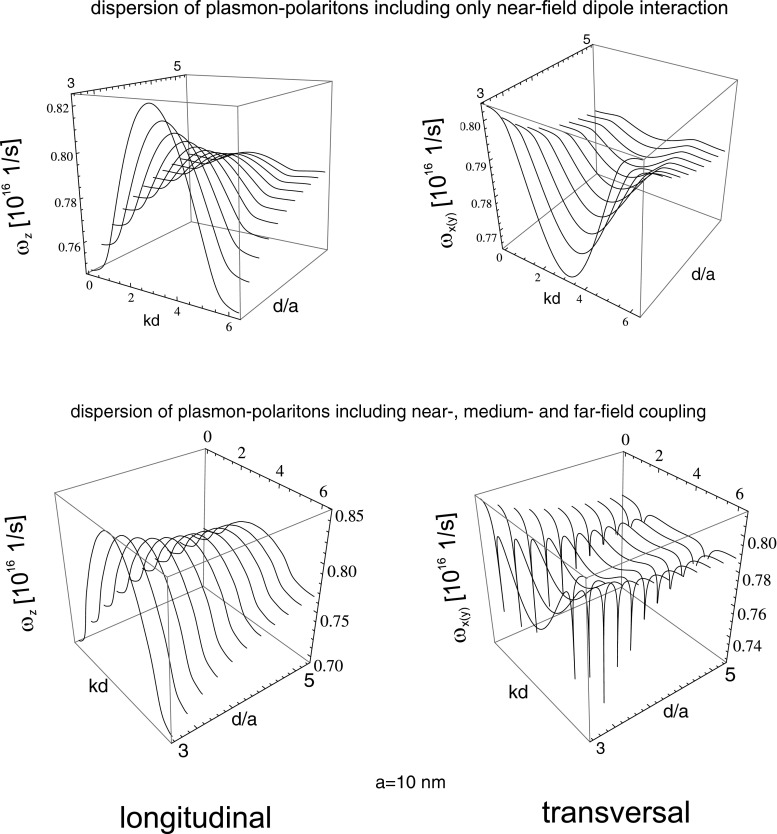
The dispersion of plasmon polaritons in the chain for both polarizations; with inclusion of only near-field coupling (*upper*), and with all near-, medium-, and far-field coupling (*lower*)

**Fig. 4:**
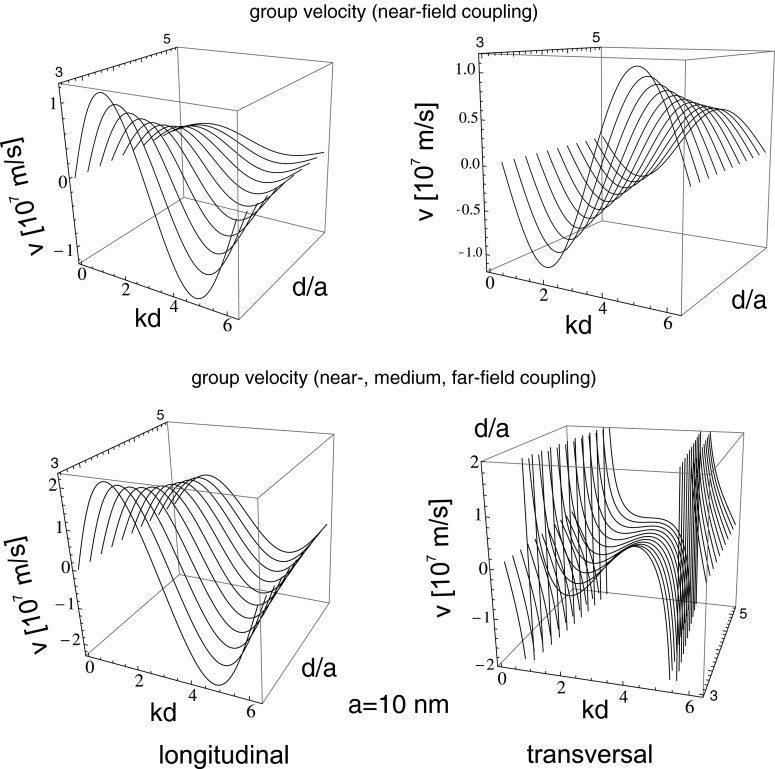
The group velocity for both polarizations; with inclusion of only near-field coupling (*upper*), and with all near-, medium-, and far-field coupling (*lower*)

In this way, the propagation of plasmon polaritons along the discrete metallic nano-structure (the chain) resembles a well known phenomenon of plasmon polariton on the 2D interface between metal and dielectric [6–8]. The radiatively undamped propagation of plasmon polariton along the chain is a similar behavior, associated with concentration of e–m energy along the chain and with the group velocity ca. one order lower than *c*. All these properties are illustrated in Figs. 2, 3, and 4 (for parameters listed in Table 1). The metallic nano-chain behaves thus like an ideal wave-guide for plasmon polaritons suitable for arrangement of sub-diffraction circuits.

**Table 1:** Nano-sphere parameters assumed for calculation

Material	Au	
Bulk plasmon energy	$\hbar \omega _{p} $	8.57 eV
Bulk plasmon frequency	$\omega _{p}$	1.302 × 10^16^ 1/s
Mie dipole plasmon energy	$\hbar \omega _{1}$	4.94 eV
Mie frequency	$\omega _{1}=\omega _{p}/\sqrt {3}$	0.752 × 10^16^ 1/s
Constant in Eq. 5	*C*	1
Fermi velocity	$v_{F}$	1.396 × 10^6^ m/s
Bulk mean free path	$\lambda _{b}$	53 nm

One can also calculate the group velocity for both polarization modes. In approximate form, taking only the first term of quickly convergent sums with denominators $n^{3}$ and $n^{2}$, while the accurate singular far-field zone term, one can rewrite the dispersion relations (56) in Appendix as follows:

33 $$\begin{array}{rll} \omega_{z} &=&\omega_{1}\left( 1- 4 \frac{a^{3}}{d^{3}}cos(kd) cos(\omega_{1} d/c)\right.\\ &&\qquad\; \left.-4 \frac{a^{2}}{d^{2}} \left(\frac{\omega_{1} a}{c}\right) cos(kd) sin(\omega_{1} d/c)\right)^{1/2}, \end{array} $$

34 $$\begin{array}{rll} \omega_{x(y)}&=&\omega_{1}\left( 1+2 \frac{a^{3}}{d^{3}} cos(kd) cos(\omega_{1} d/c)\right.\\ &&\qquad + 2 \frac{a^{2}}{d^{2}} \left(\frac{\omega_{1} a}{c}\right)cos(kd) sin(\omega_{1} d/c)+\frac{a}{d} \frac{1}{2}\left(\frac{\omega_{1} a}{c}\right)^{2}\\ &&\qquad \times~ ln\big[4\left(1-cos(kd+\omega_{1} d/c)\right)\\ &&\qquad\;\times\left. \left(1-cos(kd-\omega_{1} d/c)\right.\big]{\vphantom{\omega_{x(y)}=\omega_{1}\left( 1+2 \frac{a^{3}}{d^{3}}\right.}} \right)^{1/2}. \end{array} $$

The group velocity attains thus the form,

35 $$ v_{z} = \frac{\partial \omega_{z}}{\partial k} =\omega_{1} \frac{2\frac{a^{3}}{d^{2}} sin(kd)cos(\omega_{1} d/c) +2 \frac{a^{2}}{d} \left(\frac{\omega_{1} a}{c}\right) sin(kd) sin(\omega_{1} d/c)} {\sqrt{1- 4 \frac{a^{3}}{d^{3}}cos(kd) cos(\omega_{1} d/c) -4 \frac{a^{2}}{d^{2}} \left(\frac{\omega_{1} a}{c}\right) cos(kd) sin(\omega_{1} d/c)}} $$

36 $$\begin{array}{lll} &&v_{x(y)} = \frac{\partial \omega_{x(y)}}{\partial k}=\omega_{1} \frac{{\cal{E}}} {\sqrt{{\cal{F}}}},\\ &&{\cal{E}}=-\frac{a^{3}}{d^{2}} sin(kd) cos(\omega_{1} d/c)\\ &&\quad -\frac{a^{2}}{d}\left(\frac{\omega_{1} a}{c}\right) sin(kd) sin(\omega_{1} d/c)+\frac{a}{2} \left( \frac{\omega_{1} a}{c}\right)^{2}\\ &&\quad\times\left[\frac{sin(kd +\omega_{1} d/c)}{1-cos(kd+\omega_{1} d/c)} + \frac{sin(kd -\omega_{1} d/c)}{1-cos(kd-\omega_{1} d/c)}\right],\\ &&{\cal{F}}{\kern-2.5pt}={\kern-2.5pt}1+2 \frac{a^{3}}{d^{3}} cos(kd) cos(\omega_{1} d/c) \\ &&\quad + 2 \frac{a^{2}}{d^{2}} \left(\frac{\omega_{1} a}{c}\right)cos(kd)sin(\omega_{1} d/c)+\frac{a}{d} \frac{1}{2}\left(\frac{\omega_{1} a}{c}\right)^{2}\\ &&\quad\times~ ln[4(1{}-{}cos(kd{}+{}\omega_{1} d/c))(1-cos(kd-\omega_{1}d/c))]. \end{array} $$

The above expressions substitute Eq. 21 when besides the near-field zone also medium- and far-field zone contributions are included. Worth noticing is the hyperbolic singularity in the group velocity formula for transversal polarization, induced by logarithmic singularity due to far-field zone constructive interference of fields of all particles in the chain. This local increase of the velocity is probably responsible for indicated previously long-range fainting mode of plasmon polariton propagation in scenario when a selected single nano-sphere is excited [12, 13, 19]. In that case, the numerical analysis indicated the long-range signal. Remarkably, the damping rate was not small for this singular mode. According to our approach in the singular point of dispersion, the corresponding damping rate is not singular but rapidly grows (discontinuous finite jump), cf. Fig. 2. Thus one can suspect that numerically noticed long-range propagation of local modes corresponds to group velocity enhancement, as presented in Fig. 3 (the range of signal propagation is of order of damping time multiplied by group velocity of a particular mode). As the singular point is isolated, the corresponding mode is fainting and probably impossible to excite in practice, since for each realistic wave, packet summation of contribution of both sides of the hyperbolic singularity will cancel themselves, reducing, in that manner, the local increase of the packet velocity. This seems to be in compliance with experiment, where wave packets not too sharp abrupt in wave vector space are attainable only. The above analysis of the isolated logarithmic singularity in the transversal modes explain also an observation [12] that a finite length of the chain quenches the long-range propagating mode, which is clear as the finite sum is not divergent and reduces local increase of the group velocity. Simultaneously all other details of dispersion and of damping rate for both polarizations are robust against shortening the chain, which agrees with other numerical studies [9, 11]. Nevertheless, again for the transversal mode, the exact quenching of irradiation losses requires contribution of infinite number of far-field zone terms. Nevertheless, even for relatively short chain of ca. 10 nano-spheres, only small discrepancies occur in the vicinity of boundaries of nonradiative range defined by $kd-\omega _{1} d/c=0$ and $kd+\omega _{1} d/c =2\pi $. For other details, the difference between infinite number of terms and only of ca. 10 terms included is negligible.

**Fig. 5:**
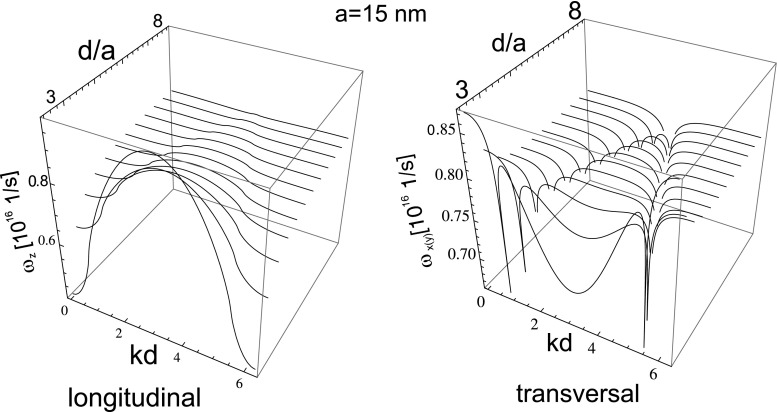
Flattening of energy dispersion with the chain separation growth; for $d/a$ exceeding ca. 6, only features are singularity lines for transversal band repeating due to periodicity; with growing *a* the flattening begins at larger $d/a$

With regard to the group velocity, one can observe its dependence of the chain geometry—the nano-sphere radius and the chain separation. With growth of the radius *a*, the amplitude of the velocity also grows while diminishes with enhancement of the separation *d*. For $d/a$ exceeding ca. 8, the energy dispersion is almost flat and in this band landscape, the only features are singular lines for transversal modes (in the coordinates *kd* and $d/a$) repeating due to periodicity, cf. Fig. 5. For such flat bands, the group velocity is almost zero except of singularity points vicinity. This explains the numerical observation [12] that the long-range modes manifest themselves especially distinctly in the case of large separation in the chain.

### Nonlinear Corrections to the Lorentz Friction

Besides the main contribution to the Lorentz friction field (6), there are some small nonlinear corrections to this field, which turn out to be important in collective plasmon propagation in metallic arrays.

For a metallic nano-sphere located (the center) in $\mathbf {R}_0$, the electric dipole of electrons (fluctuation of electron density beyond the uniform distribution compensated by positive jellium) equals to,

37 $$ \mathbf{D}(\mathbf{R}_{0},t)=e\int_{V}\delta \rho (\mathbf{r},t)\mathbf{r}d^{3}r. $$

This dipole corresponds to surface plasmons which oscillates with Mie frequency $\omega _{1}=\omega _{p}/\sqrt {3}$, where $\omega _{p}$ is bulk plasmon frequency. These plasmons are not everlasting excitations and are damped due to scattering phenomena with the damping rate, $\frac {1}{\tau _{0}}=\frac {v_{F}}{2a}+\frac {Cv_{F}}{2\lambda _{b}}$ and due to irradiation losses. For large nano-spheres, the most effective mechanism of plasmon damping is related to the irradiation energy losses, which, as described above, for the case of irradiation to far-field zone can be expressed by the Lorentz friction [24, 26].

Assuming quasiclassically that electrons in the nano-sphere have positions $\mathbf {r}_{i}$ and assuming static jellium, the dipole of the nano-sphere, $\mathbf {D}(\mathbf {R}_{0},t)\hspace *{-2pt}=\hspace *{-2pt}e\hspace *{-2pt}\sum _{i\hspace *{-0.5pt}=\hspace *{-0.5pt}1}^{N_{e}} \mathbf {r}_{i}\hspace *{-2pt}=\hspace *{-2pt}eN_{e}\mathbf {r}_{e}(t)$, where $\mathbf {r}_{e} =\sum _{i=1}^{N_{e}}\mathbf {r}_{i}/N_{e}$ is the mass center of the electron system, $N_{e}$ is the number of electrons in the nano-sphere. In the case of dynamics, the velocity of the mass center equals to, $\mathbf {v}_{e}=\sum _{i=1}^{N_{e}}\mathbf {v}_{i}/N_{e}$.

In order to determine nonlinear corrections to the formula (6) one can write out a Lorentz friction force acting on the charge $eN_{e}$ located in the mass center $\mathbf {r}_{e}(t)$, expressed in an invariant form [26],

38 $$ \mathbf{f}_{L}=\frac{2}{3}(eN_{e})^{2}\left[\frac{d^{2}\mathbf{u}}{ds^{2}}-\mathbf{u}\left(\frac{dU_{j}}{ds}\right)^{2}\right],\;\;j=1,...,4 $$

where $ds=cdt\sqrt {1-v_{e}^{2}/c^{2}}$,

$$ U_{j}=\left\{ \begin{array}{l}\mathbf{u}=\mathbf{v}_{e}/\Big(c\sqrt{1-v_{e}^{2}/c^{2}}\Big)\\ u_{4}=i/\sqrt{1-v_{e}^{2}/c^{2}}\\\end{array}\right. ,\;\; U_{j}^{2}=-1. $$

Up to terms of order $v_{e}^{2}/c^{2}$ with respect to the main term, one can write the electric field equivalent to the Lorentz friction force,

39 $$\begin{array}{rll} \mathbf{E}_{L}(t) &=& \frac{\mathbf{f}_{L}}{eN_{e}} =\frac{2}{3}(eN_{e})\frac{1}{c^{3}}\\ && \times\left\{\frac{d^{2}\mathbf{v}_{e}}{dt^{2}}+\frac{1}{c^{2}}\left[\frac{3}{2}\frac{d^{2}\mathbf{v}_{e}}{dt^{2}} v_{e}^{2}+3\frac{d\mathbf{v}_{e}}{dt}\right.\right.\\ &&\qquad\left.\left.\times\left(\mathbf{v}_{e}\cdot \frac{d\mathbf{v}_{e}}{dt}\right)+\mathbf{v}_{e}\left(\mathbf{v}_{e}\cdot \frac{d^{2}\mathbf{v}_{e}}{dt^{2}}\right)\right]\right\}.\\ \end{array} $$

Next, using dimensionless variables, $t'=t\omega _{1}, \mathbf {R}(t')=\frac {\mathbf {r}_{e}(t)}{a}, \dot {\mathbf {R}}(t')=\frac {d\mathbf {r}_{e}(t)}{a\omega _{1}dt}=\frac {\mathbf {v}_{e}}{a\omega _{1}},\ddot {\mathbf {R}}(t')= \frac {d^{2}\mathbf {r}_{e}(t)}{a\omega _{1}^{2}dt^{2}}=\frac {d\mathbf {v}_{e}}{a\omega _{1}^{2}dt},$ $ \stackrel {...}{\mathbf {R}}(t')=\frac {d^{2}\mathbf {v}_{e}(t)}{a\omega _{1}^{3}dt^{2}} $, (dots indicate derivatives with respect to *t’*), one can write out the dynamical equation in a convenient form. Taking into account that the dipole corresponding to surface plasmons,

40 $$ \mathbf{D}=eN_{e}a\mathbf{R} $$

satisfies equation of oscillatory-type, one can write out this equation in the following form (incorporating also the Lorentz friction force),

41 $$ \begin{array}{rlll} &&\stackrel{..}{\mathbf{R}}+\stackrel{}{\mathbf{R}}+\frac{2}{\tau_{0}\omega_{1}}\stackrel{.}{\mathbf{R}}=\frac{2}{3}\left(\frac{\omega_{p} a}{\sqrt{3}c}\right)^{3}\left\{\stackrel{...}{\mathbf{R}}\right.\\&&\quad \left.+\left(\frac{\omega_{p} a}{\sqrt{3}c}\right)^{2}\left[\frac{3}{2}\stackrel{...}{\mathbf{R}} (\stackrel{.}{\mathbf{R}}\cdot \stackrel{.}{\mathbf{R}})+3\stackrel{..}{\mathbf{R}}(\stackrel{.}{\mathbf{R}}\cdot \stackrel{..}{\mathbf{R}})+\stackrel{.}{\mathbf{R}}(\stackrel{.}{\mathbf{R}}\cdot \stackrel{...}{\mathbf{R}})\right]\right\},\\\end{array} $$

the terms on r.h.s. of the above equation describe the Lorentz friction including relativistic nonlinear corrections (in bracket) beyond the ordinary main linear term $\sim \stackrel {...}{\mathbf {R}}$, as previously given by (6).

For the case when $\frac {1}{\tau _{0}\omega _{1}},\;\left (\frac {\omega _{pa}}{c\sqrt {3}}\right )^{3}\ll 1$ (well-fulfilled for nano-spheres with radii 52 – 50 nm, Au or Ag), one can apply perturbation method of solution, and one can assume $\ddot {\mathbf {R}}+\mathbf {R}=0$ in zero-order perturbation. In the next step of perturbation, one can thus substitute $\stackrel {..}{\mathbf {R}}=-\stackrel {}{\mathbf {R}}$ and $\stackrel {...}{\mathbf {R}}=-\stackrel {.}{\mathbf {R}}$ in the r.h.s. of the Eq. 41.

#### Nonlinear Correction to Plasmon Radiation Losses of Single Nano-sphere

Let us consider first a single metallic nano-sphere with dipole-type surface oscillations with the dipole $\mathbf {D}$. In the framework of the perturbation method of solution of dynamical equation of oscillatory type for the dipole, Eq. 41, in the first order of perturbation, attains the following form (including the damping of plasmons due to scattering with the rate $\frac {1}{\tau _{0}}$ and due to radiation losses accounting for the linear term of Lorentz friction, while the r.h.s. of the Eq. 42 expresses nonlinear corrections to Lorentz friction),

42 $$\begin{array}{l} \ddot{\mathbf{R}}+\mathbf{R}+\left[\dfrac{2}{\tau_{0} \omega_{1}}+\dfrac{2}{3}\left(\dfrac{\omega_{p} a}{\sqrt{3}c}\right)^{3}\right]\dot{\mathbf{R}} \\ \quad =\dfrac{2}{3}\left(\dfrac{\omega_{p} a}{\sqrt{3}c}\right)^{5}\left\{-\dfrac{5}{2}\dot{\mathbf{R}}(\dot{\mathbf{R}}\cdotp\dot{\mathbf{R}})+3\mathbf{R}(\dot{\mathbf{R}}\cdotp\mathbf{R})\right\}. \end{array} $$

**Fig. 6:**
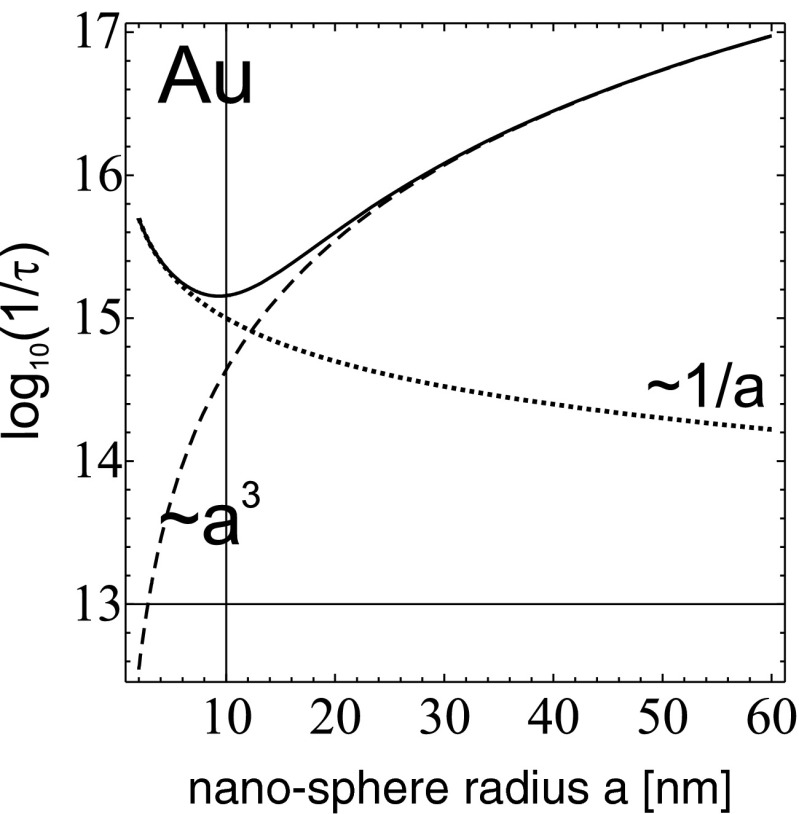
Contributions to the damping rate of surface plasmon oscillations in the nano-sphere versus the nano-sphere radius, including the scattering attenuation ($\sim v_{F}/(2\lambda _b)+ C v_{F}/(2a)$) (*dotted line*) and the Lorentz friction damping ($\sim a^{3}$) (*dashed line*); for radii greater than ca 15 nm the second channel dominates in overall damping (*upper line*); the logarithmic scale for attenuation ratio has been used

The above nonlinear differential equation can be solved by application of the asymptotic method, as described in Ref. [34]. According to this method, one can find the solution of Eq. 42 in the following form ($\mathbf {R}=R\frac {\mathbf {r}}{r}$),

43 $$ R(t)=\dfrac{A_{0} e^{-\dfrac{t}{\tau}}}{\sqrt{1+\frac{9}{8}\gamma {A_{0}}^{2}\left(1-{e}^{-\frac{{2t}}{\tau}}\right)}}\cos(\omega_{1} t+\theta_{0}), $$

where $A_{0}$ and $\theta _{0}$ are adjusted to initial conditions, and $\frac {1}{\tau \omega _{1}}=\frac {1}{\tau _{0} \omega _{1}}+\frac {1}{3}\left (\frac {\omega _{p} a}{\sqrt {3}c}\right )^{3}\approx \frac {1}{3}\left (\frac {\omega _{p} a}{\sqrt {3}c}\right )^{3}$ (what is satisfied for *a* larger than ca. 15 nm), $\gamma =\tau \omega _{1}\frac {1}{3}\left (\frac {\omega _{p} a}{\sqrt {3}c}\right )^{5}.$ From the form of equation for $\frac {1}{\tau \omega _{1}}$ (below the Eq. 43) it follows that $\frac {1}{\tau \omega _{1}}$ is always positive. The scattering term, $\frac {1}{\tau _{0}}=\frac {Cv_{F}}{2a}+\frac {v_{F}}{2\lambda _{b}}$, is negligible (for nano-sphere radius beyond ca. 15 nm) in comparison with the linear contribution of the Lorentz friction, as it is demonstrated in Fig. 6.

The scale of the nonlinear corrections is given by the coefficient $\gamma \approx 7.3 \times 10^{-4} (a[nm])^{2}$. As this coefficient is small, one can neglect the related contribution in the denominator of the solution (43), which results in ordinary linear solution of damped oscillations. It means that the nonlinear corrections to the Lorentz friction have no significance in the case of plasmon oscillations of a single nano-sphere. This situation changes, however, in the case of collective plasmon excitation propagating along the metallic nano-chain, as it will be described in the following paragraph.

### Nonlinear Correction to Radiation Loses of Plasmon Polariton in the Nano-Chain

In the case of dynamics of plasmon polaritons in the metallic nano-chain, inclusion of nonlinear correction to the Lorentz friction resolves itself to accounting of these nonlinear contributions in the Eq. 16 via the formula for $E_{L}$. Instead of Eq. 18 we get thus the following equation (the Lorentz friction term was represented similarly as in Eq. 42),

44 $$\begin{array}{rll} &&\ddot{R}_{\alpha}(k_{\alpha},t\omega_{1})+ \tilde{\omega}_{\alpha}^{2} R_{\alpha}(k_{\alpha},t\omega_{1})\\ &&=-2\dot{R}_{\alpha}(k_{\alpha},t\omega_{1})\left\{\frac{1}{\tau_{\alpha} \omega_{1}} + \right. \frac{1}{3}\left(\frac{\omega_{p}a}{c\sqrt{3}}\right)^{5}\\ &&\qquad \times~\left.\left[\frac{5}{2}|\dot{R}_{\alpha}(k_{\alpha},t\omega_{1})|^{2}-3|R_{\alpha}(k_{\alpha},t\omega_{1})|^{2}\right]\right\}, \end{array} $$

with the renormalized frequency and damping rate given by Eq. 56 and Eq. 57, respectively (cf. Appendix for derivation).

Applying the same asymptotic methods [34] for solution of the nonlinear Eq. 44 as in the former paragraph, one can find the corresponding solutions for both regions with positive and negative damping rate, respectively.

For the positive damping rate, $ \frac {1}{\tau _{\alpha }\omega _1}>0$ (cf. Ref. [34]),

45 $$\begin{array}{rll} R_{\alpha}(k,t)&=&\dfrac{A_{\alpha 0}e^{-\dfrac{t}{\tau_{\alpha}}}}{\sqrt{1+\gamma_{\alpha}A_{\alpha 0}^{2}\left(1-e^{-\dfrac{2t}{\tau_{\alpha}}}\right)}}\cos(\omega_{\alpha}t+\theta_{0}),\\ R_{\alpha}(k,t)&\rightarrow&_{(t\rightarrow \infty)} 0\end{array} $$

where $\gamma _{\alpha }=|\tau _{\alpha }\omega _{1}|\left (\frac {\omega _{1} a}{c}\right )^{5}\frac {1}{4}\left (\frac {5}{2}\tilde {\omega }_{\alpha }^{2}-1\right )$, $\theta _{0}=kld +\phi _{0}$, $\phi _{0}$ and $A_{\alpha 0}$ are adjusted to initial conditions. We note from the form of Eq. 45 that this is a damped mode vanishing at longer time scale.

Nevertheless, for the negative damping rate, $\frac {1}{\tau _{\alpha }\omega _{1}}<0$, the solution has a different form (cf. Ref. [34],

46 $$ \begin{array}{rll} R_{\alpha}(k,t)&=&\dfrac{A_{\alpha 0}e^{\dfrac{t}{|\tau_{\alpha}|}}}{\sqrt{1+\gamma_{\alpha}A_{\alpha 0}^{2}\left(e^{\dfrac{2t}{|\tau_{\alpha}|}}-1\right)}}\cos(\omega_{\alpha}t+\theta_{0}),\\ R_{\alpha}(k,t)&\rightarrow&_{(t \rightarrow \infty)}\dfrac{1}{\sqrt{\gamma_{\alpha}}}\cos(\omega_{\alpha} t+\theta_{0})\end{array} $$

$\theta _{0}=kld +\phi _{0}$. This solution is stable; it corresponds to an undamped mode which stabilizes on the fixed amplitude, $\frac {1}{\sqrt {\gamma _{\alpha }}}$, at longer time scale, independently of initial condition expressed by $A_{\alpha 0}$.

The corresponding dipole oscillations attain in the latter case the form of monochromatic waves propagating along the chain in both directions,

47 $$ D_{\alpha}=\dfrac{e N_{e} a}{\sqrt{\gamma_{\alpha}}}\dfrac{1}{2} cos(\omega_{\alpha}t \mp kld +\phi_{0}). $$

From the above discussion, it follows that for positive attenuation rate, we deal with ordinary damped plasmon polariton propagation, not strongly modified in comparison to linear theory (due to small value of the factor $\gamma _{\alpha }$). Nevertheless, in the case of negative damping rate, the solution behaves differently—on longer time scale, this solution stabilizes on the constant amplitude independently of initial conditions. This property characterizes undamped propagation of plasmon polariton along the chain. It should be, however, noted that the existence of undamped modes in the system with scattering losses, thus with energy dissipation, would contradict energy conservation. Nevertheless, if one assumes that the system is energetically supplied by the external source synchronic to $\dot {\mathbf {R}}$, then the relatively small strength would prevail scattering losses and in the whole region of radiative losses quenching (the white region in the Fig. 2 left) the total damping rate would be negative. In such a case, we would deal with two types of plasmon polaritons: ordinary damped modes with still positive overall attenuation rate (the shaded region in the Fig. 2, additionally slightly diminished close to the borders with the white region, due to shift caused by the external energy supply) and the second one consisted of undamped modes. The modes from the first region will extinguish after the distance of order of the attenuation time multiplied by the group velocity, but the modes from the second region will continue stable propagation with fixed amplitude independently of initial conditions. These latter modes express instability (induced in this case) of the system, thus energy for this propagation is not the initial excitation energy but is the energy supplied by the pumping force. In other words, the continuous losses of plasmon oscillation energy due to scattering are instantly recovered by the external pumping. When this income prevails losses, then the packet of corresponding modes propagates without damping. This behavior is typical for other nonlinear oscillation systems, and its existence also for plasmon polaritons would be of practical significance.

## Conclusions

As indicated above, radiatively undamped modes of propagation of collective surface plasmons seem to match with experimentally observed long-range propagation of plasmon excitations along the finite metallic nano-chains [2, 4, 6, 23]. We have demonstrated the utilization of RPA semiclassical model of plasmon oscillations in metallic nano-spheres to description of collective surface plasmon polariton propagation along metallic nano-chain. The oscillatory form of dynamics both for volume and surface plasmons, rigorously described upon the RPA semiclassical limit, fits well with the large nano-sphere case, with radii of several to several tens of nanometers, what is confirmed also by experimental observations. The most important property of plasmons on large nano-spheres is the very strong e–m irradiation caused by these excitations, which results in quick damping of oscillations. The attenuation effects for plasmons were not, however, included into the quantum RPA model. Nevertheless, they could be included in a phenomenological manner, taking advantage of the oscillatory form of dynamical equations. Some information on plasmon damping can be taken from microscopic analysis of smaller metallic clusters, with size of 1 – 2 nm (especially made by LDA and TDLDA methods of numerical simulations employing Kohn–Sham equation). For larger nano-spheres, these effects, mainly of scattering type (also Landau damping), are, however, of lowering significance diminishing with radius growth, as $\frac {1}{a}$, while the damping of plasmons starts to be dominated by irradiation losses growing as $a^3$.

The irradiation effects overwhelming the energy losses in the case of large nano-spheres can be grasped in terms of the Lorentz friction, which reduces the charge movement. This approach has been analyzed in the present paper. Two distinct situations were indicated, the first one—of the free radiation to far-field zone in dielectric (or vacuum) surroundings of single nano-particle and the second one, when in the near-field zone of plasmons an additional charged system is located.

This additional system of charges acting as the e–m energy receiver in vicinity of the metallic nano-sphere with plasmons, strongly modifies the energy balance of the source and in this way modifies energy emission in comparison to the free emission in vacuum or in dielectric surroundings. In particular, the Lorentz friction is modified in the case of energy receiver presence in the near-field zone of plasmons, in comparison to simple free emission to the far-field zone. The e–m energy receiver located close to emitting nano-sphere could be a semiconductor (as in the case of metallic modified solar cells) or other metallic nano-spheres (as in the case of metallic nano-chain). The latter situation has been analyzed in this paper. We have shown previously within the near-field coupling approximation [22] that along the infinite nano-chain, the collective plasmon polaritons can propagate (being collective surface plasmons coupled by e–m field in near-field zone), which at certain values of nano-sphere radius and separation in the chain, occur to be undamped modes. Simultaneously, the instability regions of linear theory of plasmon polariton dynamics occur, which shows that some corrections must be included to assure energy conservation.

In this paper, we have examined this artifact of near-field zone coupling and have demonstrated that inclusion of medium-field and far-field contributions removes the instability and leads to the typical for plasmon polaritons exact quenching of radiative losses. We have analyzed corrections due to medium- and far-field zone contributions in full retarded form, including logarithmic isolated singularity in dispersion of transversal modes due to constructive interference effect of far-field dipole term. The dispersion, damping rate, and group velocity for longitudinal and transversal polarizations with respect to the chain orientation were analyzed in dependence on the nano-sphere size and separation in the chain. The results explain some previous, numerical type observations related to existence of fainting far-range mode of plasmon polaritons due to far-field constructive interference (described by Markel and Sarychev). The wave packet of single-point excitation in the chain includes all wave vectors (as Fourier picture of point like initial signal), also from the region of hyperbolic singularity of group velocity for transversal polarization. As this singularity is isolated and providing opposite sign contributions of its both sides, the resulting far-range propagation (related to group velocity growth) corresponds to the fainting mode, though with higher velocity and thus larger range in comparison to other packet components. The manifestation of this behavior is better visible for larger separation of nano-spheres in the chain when the group velocity out of singularity is strongly lowered due to flattening of the spectrum, which also fits to previous numerical observations.

We have examined also a nonlinear corrections to collective plasmon polariton dynamics along the chain, including nonlinear contribution to Lorentz friction force. Even though the related nonlinearity is small, it suffices to regularize the instable linear approach in the case of external energy supply. We noted the presence in this case of undamped excitations, which have fixed amplitude independently how small or large the initial conditions were. This excitations, typical for nonlinear systems, would have some practical significance, e.g., to enhance resolution of photon sensors with coverings of plasmon nano-systems providing long-range collective plasmon polariton modes in widen range of frequencies depending on chain parameters.

## Figures and Tables

**Fig. 7:**
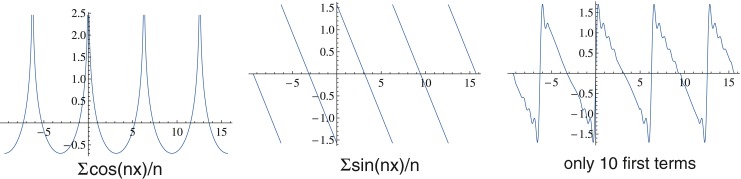
The sum $\sum \limits _{n=1}^{\infty }\frac {cos(nx)}{n}=-\frac {1}{2} ln[2(1-cos(x))] (left), \sum \limits _{n=1}^{\infty }\frac {sin(nx)}{n}= \frac {\pi -x}{2}, \; for\; 0<x<2\pi $ (center), the same sum with only 10 first terms (right)

**Fig. 8:**
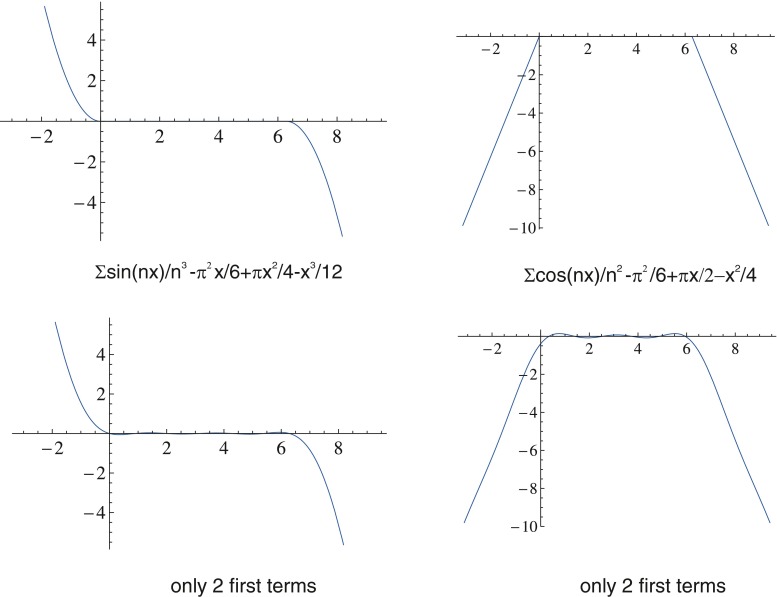
The sum $\sum \limits _{n=1}^{\infty }\frac {sin(nx)}{n^{3}} -\frac {\pi ^{2} x}{6}+\frac {\pi x^{2}}{4}-\frac {x^{3}}{12} (left), \sum \limits _{n=1}^{\infty }\frac {cos(nx)}{n^{2}} -\frac {\pi ^{2}}{6}+\frac {\pi x}{2} +\frac {x^{2}}{4} $ (right); the same sums with only two first terms included (bottom)

## Appendix: Dispersion and Attenuation of Plasmon Polariton in the Chain for Complete Dipole Interaction Dipole and the Derivation of Eq. 44

In the dipole interaction approximation, the Fourier component of the electric field is given by Eq. 13. Assuming ${\mathbf r}$ as the position of the sphere (center) irradiating e–m energy due to its dipole surface plasmon oscillations $\mathbf {D}(\mathbf {r},t)$, and ${\mathbf r}_0$ as the position of another sphere (center), with respect to the center of the former one, the field in this point ${\mathbf E}({\mathbf r}, {\mathbf r}_0, t)$ is given by Eq. 13 with ${\mathbf n}_0={\mathbf r}_0/r_0$. In the case of a linear chain, both vectors ${\mathbf r}$ and ${\mathbf r}_0$ are collinear and oriented along the chain (*z*–axis). If the coordinate origin coincides with the center of one sphere in the chain, for the *l*th sphere located in the point ${\mathbf r}_l=(0,0,ld)$, the electric field caused by neighboring spheres located in points ${\mathbf r}_m=(0,0,md)$, $m\neq l$ must be included (in the case of the equidistant chain, $r_m=|m|d$ and $r_{ml}=|l-m|d$) and the equation for the surface plasmon oscillations of the *l*th sphere attains the form,

48 $$\begin{array}{lll} &&\left[\frac{\partial^{2}}{\partial t^{2}}+ \frac{2}{\tau_{0}} \frac{\partial}{\partial t} +\omega_1^{2}\right]D_{\alpha}(ld,t)\\ &&=\omega_{1}^{2}a^{3} \sum\limits_{m=-\infty, \;m\ne l }^{m=\infty} E_{\alpha}\left(md,t-\frac{|l-m|d}{c}\right) +\omega_{1}^{2}a^{3} E_{L\alpha}(ld,t).\end{array} $$

Changing to variables $t\omega _{1}$, $R_{\alpha }(ld,t\omega _1)= \frac {D_{\alpha }(ld,\omega _{1}t/\omega _{1})}{eN_{e} a}$, and using the explicit form for the Lorentz friction term, $E_{L\alpha }$, one can rewrite Eq. 48 in the form (dot indicates the derivative with respect to $t'=t\omega _1$, note also that $R(t\omega _{1})\sim e^{\omega t}=e^{\frac {\omega }{\omega _{1}} \omega _{1} t}$),

49 $$\begin{array}{rll} &&\ddot{R}_{\alpha}(ld,t\omega_{1})+ {R}_{\alpha}(ld,t\omega_{1})\\ &&\quad = a^{3}\sum\limits_{m=-\infty,{\kern1pt} m\neq l}^{\infty}\left\{ R_{\alpha}\left(md, t\omega_1-\frac{\omega_{1}|l-m|d}{c}\right)\right.\\ && \qquad \times\left( \frac{(\omega /c)^{2}}{|l-m|d}+\frac{i\omega /c}{(|l-m|d)^{2}}-\frac{1}{(|l-m|d)^{3}}\right)\\ && \qquad + \delta (\alpha,z) R_{\alpha} \left(md, t\omega_{1}-\frac{\omega_{1}|l-m|d}{c}\right)\\ && \qquad \times\left. \left( -\frac{(\omega /c)^{2}}{|l-m|d}-\frac{i3\omega /c}{(|l-m|d)^{2}}+\frac{3}{(|l-m|d)^{3}}\right)\right\}\\ &&\qquad -\frac{2}{\tau_{0}\omega_{1}}\dot{R}_{\alpha}(l d,t\omega_{1})+\frac{2}{3}\left(\frac{\omega_{p}a} {c\sqrt{3}}\right)^{3}\\ &&\bigg\{\stackrel{...}{R}_{\alpha}(ld,t\omega_{1})+\left(\frac{\omega_{p}a}{c\sqrt{3}}\right)^{2}\bigg[\frac{3}{2}\stackrel{...}{R}_{\alpha}(ld,t\omega_{1}) |\dot{R}_{\alpha}(ld,t\omega_{1})|^{2} \\ &&\qquad +3 \ddot{R}_{\alpha}(ld,t\omega_{1})|\dot{R}_{\alpha}(ld,t\omega_{1}) \ddot{R}_{\alpha}(ld,t\omega_{1})| \\ &&\qquad +\dot{R}_{\alpha}(ld,t\omega_{1})|\dot{R}_{\alpha}(ld,t\omega_{1})\stackrel{...}{R}_{\alpha}(ld,t\omega_{1}) |\bigg]\bigg\}, \end{array} $$

where $\omega _{1}=\frac {\omega _{p}}{\sqrt {3}}$ is the free surface dipole plasmon frequency in a nano-sphere. Assuming now the perturbation method of the solution of the above equation, in its r.h.s. one can substitute, $\ddot {R}_{\alpha }$ by $R_{\alpha }$ (as for free oscillations). Taking advantage from the periodicity of the infinite chain, one can consider the solution of the above equation in the Bloch-type form,

50 $$ R_{\alpha}(ld,t)=R_{\alpha}(k,t)e^{\mp ikld}, $$

$0\leq kd <2\pi $. Thus the contribution to Eq. 49 from other nano-spheres can be rewritten as,

51 $$\begin{array}{rll} &&e^{\mp ikld} e^{-i\omega t -t/\tau} R_{\alpha} (k)a^{3}\sum\limits_{n=-\infty, n\neq l}^{\infty} e^{iknd}(F_{1}(|n|)\\ &&\quad+i F_{2}(|n|)) e^{i|n|\omega d/c}\\&& = e^{\mp ikld}e^{-i\omega t -t/\tau} R_{\alpha} (k) 2a^{3}\sum\limits_{n=1, n\neq l}^{\infty} cos(knd) (F_{1}(|n|)\\ &&\quad+i F_{2}(|n|)) e^{i|n|\omega d/c}, \end{array} $$

which is Eq. 23 with *F* **1** and *F* **2** defined by Eqs. 24 and 25, respectively.

Simultaneously, the Lorentz friction term together with the scattering attenuation term attain the form,

52 $$\begin{array}{rll} &&-{}2\dot{R}_{\alpha}(ld,t\omega_1){}\left\{{\kern-1.5pt} \frac{1}{\tau_{0}\omega_1} {}+{}\frac{1}{3}{}\left(\frac{\omega_{p} a}{c\sqrt{3}}\right)^{3} +\frac{1}{3}\left(\frac{\omega_{p} a}{c\sqrt{3}}\right)^{5}\right.\\ &&\qquad \left. \times \left[\frac{5}{2}|\dot{R}_{\alpha}(ld,t\omega_1)|^{2} -3|R_{\alpha}(ld,t\omega_1)|^{2}\right]\right\}. \end{array} $$

The term (51) resolves itself to the expression (26) and next to the explicit contributions entering the real and imaginary parts of the equation for oscillations, as given by Eqs. 27–30. In this representation one can perform the summations in Eqs. 27–30. In order to perform these summations one can use the following relations,

53 $$\begin{array}{rll} cos(p) sin(q) &=&\frac{1}{2}(sin(p+q) -sin(p-q)),\\ cos(p) cos(q)&=&\frac{1}{2}(cos(p+q)+cos(p-q)), \end{array} $$

with $p=nkd$ and $q=n\omega d/c$;

54 $$\begin{array}{rll} \sum\limits_{n=1}^{\infty}\frac{sin(nx)}{n}&=&\frac{\pi -x}{2},\; for\;\; 0<x<2\pi, \\ \sum\limits_{n=1}^{\infty}\frac{cos(nx)}{n}&=&\frac{1}{2}ln \frac{1}{2(1-cos(x))},\\ \sum\limits_{n=1}^{\infty}\frac{cos(nx)}{n^{2}}&=&\frac{\pi ^{2}}{6}-\frac{\pi x}{2}+\frac{x^{2}}{4}, \; for\;\; 0<x<2\pi,\\ \sum\limits_{n=1}^{\infty}\frac{sin(nx)}{n^{3}}&=&\frac{\pi ^{2} x}{6}-\frac{\pi x^{2}}{4}+\frac{x^{3}}{12}, \; for\; \;0<x<2\pi \end{array} $$

The first and the two latter formulae hold only for $0<x<2\pi $, while the second one is exact for all *x*. From the second equation, we see that the sum $\sum \frac {cos(nx)}{n}$ has isolated divergence points (logarithmic) at $x=l2\pi $, *l* integer (cf. Fig. 7). Other sums are convergent for any value of the argument *x*. The sum $\sum \frac {sin(nx)}{n}$ is convergent at $x=l2\pi $, *l* integer, but has the finite discontinuity jump at these points (cf. Fig. 7). The sums $\sum \frac {cos (nx)}{n^{2}}$ and $\sum \frac {sin (nx)}{n^{3}}$ are convergent also outside the sector $(0,2\pi )$, though are not equal to the above given r.h.s. of the two last formulae (cf. Fig. 8). According to Eq. 53, the range for arguments, where (54) are applicable, will be given by $kd -\frac {\omega }{c} d>0$ and $kd +\frac {\omega }{c} d<2\pi $, as indicated in Fig. 2 (for $\omega =\omega _{1}$, according to the assumed next perturbation iterative method of solution). The sums with the denominator $n^{2}$ or $n^{3}$ are quickly convergent and few first terms give the almost exact value of the sum (cf. Fig. 8), while for the sums with the denominator *n* it is not so and the exact infinite summation is of order there.

To solve Eq. 49, one can assume in the retardation terms in r.h.s. of this equation, $\omega =\omega _{1}$ as for the perturbation iterative method of solution, the same one as applied earlier to the Lorentz friction, i.e., $\stackrel {...}{R}=-\dot {R}$ (note, however, that for $R\sim e^{i\omega t}$, the accurate relation is, $\frac {\partial ^{3} R}{\partial t^{3}}=-\omega ^{2} \frac {\partial R}{\partial t}$). Using the above formulae, one can rewrite Eq. 49 in the first step of iterative procedure, in the following form,

55 $$\begin{array}{lll} &&\ddot{R}_{\alpha}(k,t\omega_{1})+ \tilde{\omega}_{\alpha}^{2} R_{\alpha}(k,t\omega_{1}) \\&&=-2\dot{R}_{\alpha}(k,t\omega_{1})\left\{\frac{1}{\tau_{\alpha} \omega_{1}}+\frac{1}{3}\left(\frac{\omega_{pa}}{c\sqrt{3}}\right)^{5}\right.\\&&\qquad\qquad\qquad\qquad\left. \times\left[\frac{5}{2}|\dot{R}_{\alpha}(k,t\omega_{1})|^{2}-3|R_{\alpha}(k,t\omega_{1})|^{2}\right]\right\}, \end{array} $$

where,

56 $$ \begin{array} {rll} \tilde{\omega}_{\alpha}^{2}&=& \dfrac{\omega_{\alpha}^{2}}{\omega_{1}^{2}}=1- a^{3} \left\{\begin{array}{l}{\cal{A}},\; \alpha=z,\\{\cal{B}},\;\alpha=x(y),\\\end{array} \right.\\{\cal{A}}&=& 2\sum_{n} cos(knd) \dfrac{2}{n^{3}d^{3}} cos (n\omega_{1} d/c) \\ && +2\sum_{n} cos(knd) \dfrac{2\omega_{1}/c}{n^{2}d^{2}} sin(n\omega_{1} d/c),\\{\cal{B}}&=& 2\sum_{n} cos(knd)\left( \dfrac{(\omega_{1} /c)^{2}}{nd} - \dfrac{1}{n^{3}d^{3}}\right) cos (n\omega_{1} d/c)\\ && -2\sum_{n} cos(knd) \dfrac{\omega_{1}/c}{n^{2}d^{2}} sin(n\omega_{1} d/c).\end{array} $$

and

57 $$ \begin{array}{rll}\frac{1}{\tau_{\alpha}\omega_{1}} &=&\left\{ \begin{array}{l}\frac{1}{\tau_{0}\omega_{1}},\; for\; kd-\frac{\omega_{1}}{c}d >0, \; kd+\frac{\omega_{1}}{c} d<2\pi,\; \alpha=z,x(y)\\\frac{1}{\tau_{0}\omega_{1}}+\frac{1}{3} \left(\frac{\omega_{p} a}{c\sqrt{3}}\right)^{3}+a^{3}\left\{\begin{array}{l}{\cal{C}}, \; \alpha=z,\\{\cal{D}},\;\alpha=x(y),\\\end{array}\right.\end{array}\right.\\{\cal{C}}&=&-\sum_{n} cos(knd) \frac{2}{n^{3}d^{3}} sin(n\omega_{1} d/c) \\ &&+\sum_{n} cos(knd) \frac{2\omega_{1}/c}{n^{2}d^{2}} cos(n\omega_{1} d /c),\\{\cal{D}}&=&-\sum_{n} cos(knd) \left(\frac{(\omega_{1}/c)^{2}}{nd}-\frac{1}{n^{3}d^{3}}\right) sin(n\omega_{1} d/c)\\ && -\sum_{n} cos(knd) \frac{\omega_{1}/c}{n^{2}d^{2}} cos(n\omega_{1} d/c).\end{array} $$

In Eq. 56, one can substitute the point-divergent sum with the exact analytic form,

58 $$\begin{array}{lll} && a^{3}\sum\nolimits_{n=1}^{\infty} \frac{cos(knd) (\omega_{1}/c)^{2} cos(n\omega_{1} d/c )}{nd}\\&&=-\frac{a}{d}\left(\frac{\omega_{1} a}{c}\right)^{2}\frac{1}{4}ln\big[4(1-cos(kd+\omega_{1}d/c ))\\&&\quad \times(1-cos(kd-\omega_{1}d/c ))\big], \end{array} $$

which explicitly displays isolated logarithmic singularity in the dispersion formula for the transversal mode. Note that the longitudinal modes are without any singularity. It is linked with the absence of constructive interference of dipole radiation in far-field zone, expressed by the term with the denominator $r_0$, in the direction collinear with dipole orientation as in the case of longitudinal polarization.

The above equation is the Eq. 44 with explicit formulae for the dispersion (56) and the damping rate (57), for both polarizations and all wave vectors $kd\in [0,2\pi )$, with respect to the nano-sphere radius *a* and the chain separation *d*. Whole information on longitudinal and transversal modes of plasmon polaritons in the chain is given by these equations (i.e., Eq. 44 with Eq. 56 and Eq. 57).
